# Implementation of isopropyl alcohol (IPA) inhalation as the first-line treatment for nausea in the emergency department: practical advantages and influence on the quality of care

**DOI:** 10.1186/s12245-021-00334-z

**Published:** 2021-02-24

**Authors:** Peter Veldhuis, Maartje Melse, Nieke Mullaart

**Affiliations:** 1 Department of Emergency Medicine, OLVG Oost, Postbus 95500, 1090 HM Amsterdam, The Netherlands; 2 Department of Emergency Medicine, Dijklander Ziekenhuis, Postbus 600, 1620 AR Hoorn, The Netherlands

**Keywords:** Nausea, Isopropyl alcohol inhalation, Emergency department, Quality of care, Implementation outcome measures

## Abstract

**Background:**

Nasal inhalation of isopropyl alcohol (IPA) seems an effective anti-emetic for the symptomatic treatment of nausea in the emergency department (ED) compared to conventional anti-emetics (Ondansetron and Metoclopramide). However, it is not yet known what the practical consequences are related to the use of IPA in the ED.

**Objectives:**

The purpose of this study was to assess the practical implications for patient care associated with IPA use and to evaluate the viability of permanent implementation of IPA inhalation as a first-line therapy for nausea in the ED.

**Methods:**

We conducted a prospective, single-center implementation study comparing ED-based care for nauseated patients before (*n*=106) and after (*n*=104) the introduction of IPA. We evaluated the treatment process and cost and assessed implementation using a survey based on recommended implementation outcome measures.

**Results:**

Comparing baseline phase to implementation phase, we found a significant increase in the percentage of patients receiving nausea treatment (66.0% versus 97.1%; *p*<0.001) and a reduction in time to treatment initiation (7 versus 1 min, *p*<0.001). Additionally, IPA introduction was associated with a decrease in the administration of conventional anti-emetics (0.52 versus 0.23 administrations per patient, *p*<0.001) and a notable drop in treatment cost (€1.33 versus €0.67 per patient). Nurses were content with IPA implementation and regarded definitive implementation as feasible and sustainable.

**Conclusion:**

Implementation of IPA as the first-line nausea treatment in the ED can increase the quality of care and improve care efficiency. Definitive implementation of IPA as a first-line treatment in the ED is both viable and practically feasible.

**Trial registration:**

NTR, NL7717, Registered on March 23, 2018 - Retrospectively registered

**Supplementary Information:**

The online version contains supplementary material available at 10.1186/s12245-021-00334-z.

## Introduction

Nausea is one of the most common complaints in the emergency department (ED) and has a great variety of possible causes [1]. Not only is nausea an uncomfortable complaint, it can also cause complications like aspiration and dehydration [2]. Ideally, it is relieved by treating the underlying cause. However, a rapid symptomatic treatment is also desirable, as it takes time to diagnose the underlying disease and for its specific treatment to take effect. The most commonly used anti-emetics in the ED are Ondansetron, Metoclopramide, and Promethazine [3]. However, it is questionable whether these conventional anti-emetics provide optimal symptomatic therapy for nausea. A systematic review in 2015 showed that none of these anti-emetics is superior to placebo when treating nausea in the ED [4]. Furthermore, studies show that only half of the patients with nausea receive symptomatic treatment in the ED [3, 5].

Nasal inhalation of isopropyl alcohol (IPA) is an alternative for conventional anti-emetics. In a systematic review of patients with postoperative nausea, IPA inhalation proved to be effective in reducing nausea severity [6]. In addition, two recent ED-based randomized controlled trials show that IPA inhalation results in a significant decrease of nausea compared to placebo [7] and oral Ondansetron [8]. No significant side effects were reported in these studies.

Thus, based on the currently available literature, IPA seems an effective anti-emetic for the symptomatic treatment of nausea in the ED. In addition to these medical outcomes, IPA conceivably offers some practical advantages. Firstly, IPA is easy to apply as no intravenous access is required and its use requires few instructions. Secondly, as the IPA inhalation swabs are packed individually, patients can use them at their own discretion. Thirdly, IPA swabs are easily available in most hospitals, facilitating implementation. Lastly, IPA swabs are much cheaper than conventional anti-emetics.

As the ED is a time restrained working field with emphasis on high-quality care, cost-effectiveness, and patient autonomy, practical advantages could serve as arguments to implement IPA inhalation as a first-line therapy for nausea. However, to date, there is no evidence that IPA translates into a practical benefit for patients and ED staff. Therefore, this study investigates the practical consequences and the viability of the permanent implementation of IPA inhalation as a first-line therapy for nausea in the ED.

## Methods

### Study design

We conducted a single-center prospective implementation study, in which IPA inhalation therapy was implemented as a first-line therapy for nauseous patients in our ED. The study was conducted in an urban secondary care hospital in The Netherlands with approximately 24,000 ED visits per year. The study started with the baseline phase, during which we prospectively collected data concerning patients receiving standard ED care for nausea. The baseline phase was followed by the implementation phase, during which IPA inhalation was used to symptomatically treat nausea. The baseline phase was between March 2018 and July 2018, and the implementation phase took place between July 2018 and March 2019. Six months after the implementation of IPA, we conducted a survey amongst our ED nurses to evaluate their experiences with IPA inhalation as a first-line treatment for nauseous patients. The study was registered in The Netherlands trial register (NL7177). Ethical approval for this study was obtained from the regional ethics committee (METC Noord-Holland), and our institutional review board approved the protocol.

### Study participant selection and data collection

Our ED nurses screened all patients visiting the ED during the study period for inclusion and exclusion criteria. Inclusion criteria included adults (≥18 years) presenting to the ED and experiencing nausea. Exclusion criteria included a known allergy to IPA or conventional anti-emetics, pregnancy, inability to inhale through the nose, a reduced level of consciousness, and any other medical condition hindering following study instructions. In case a patient was identified as eligible for inclusion, written informed consent was obtained. Nausea severity was evaluated using a visual analog score (VAS). VAS-scores are a validated tool for acute and chronic pain, but can also be used for determining nausea severity [9]. Furthermore, we registered patient characteristics, the presumed cause of nausea upon ED entry, a VAS for pain, the time of inclusion, the time of initiation of symptomatic treatment (IPA or conventional anti-emetic), the total amount of used anti-emetics, the use of IPA swabs, and the definitive diagnosis as made by the treating physicians at the end of the ED visit.

### Treatment protocol

**Fig. 1:**
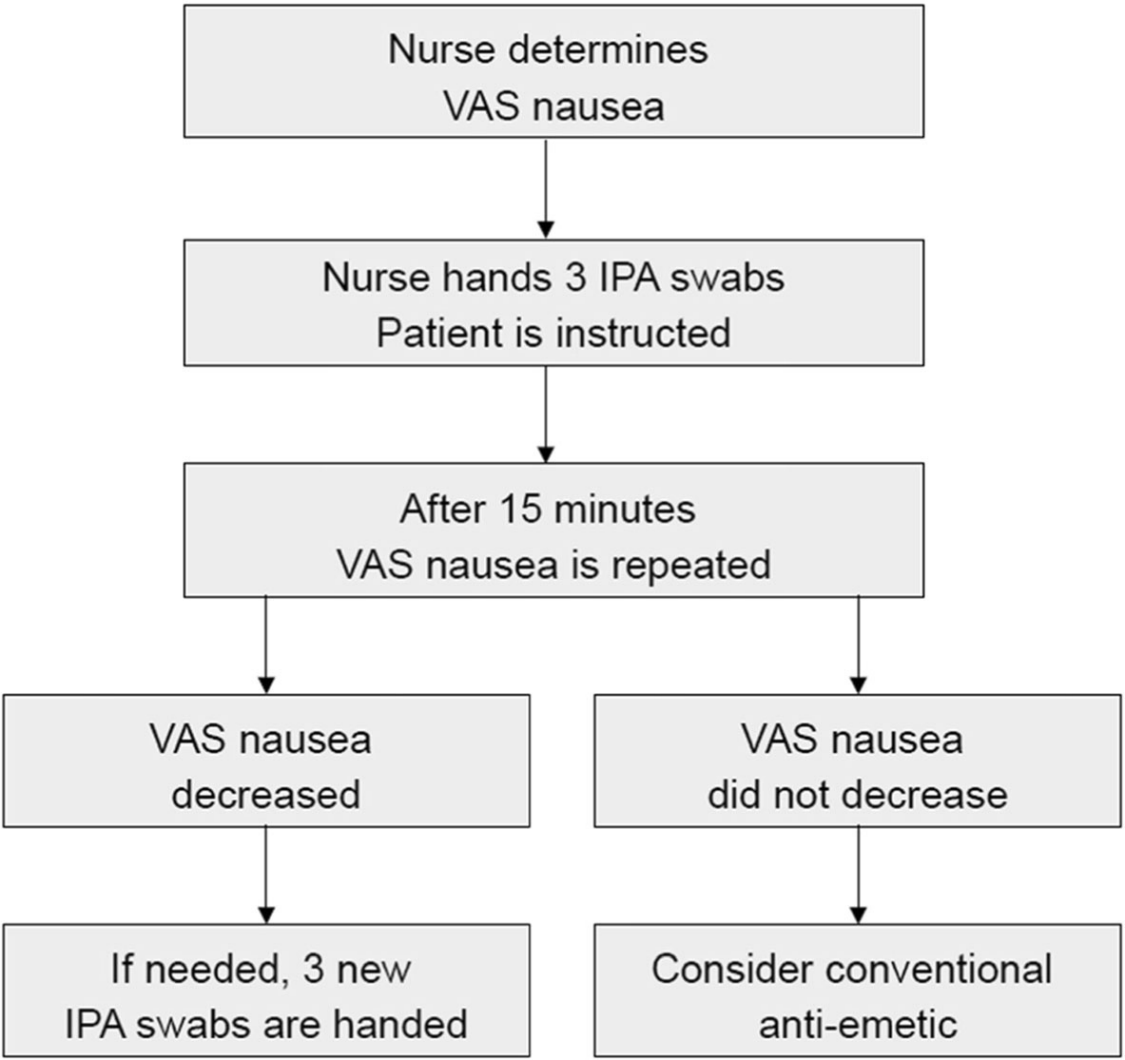
Treatment protocol for nausea during the implementation phase

In the baseline phase of the study, nauseous patients were symptomatically treated according to daily practice, and no specific treatment protocol was used. In case a patient was identified as nauseous, the nurse discussed with the treating physician if symptomatic treatment for nausea was indicated, and if so, which anti-emetic to administer. During the implementation phase, primarily the nurse decided whether symptomatic treatment for nausea was desirable, and if so, he or she provided the patient with three IPA swabs. The patients were instructed to open an IPA swab and hold it 2 cm in front of their nose while taking a number of deep nasal inhalations. Afterwards, patients could decide for themselves how frequently they used an IPA swab, with a maximum of 3 swabs every 15 min. A high level of inhalation compliance can be assumed, as the nurse was always present during the first and usually present during subsequent inhalations. Additionally, even though the instruction and practical execution of a correct application of IPA inhalation is very simple, an information sheet with inhalation instructions was present at the bedside. Fifteen minutes after the first inhalation, a new VAS for nausea was obtained. In case of a decrease in nausea, IPA therapy was continued if needed. If nausea had not changed or even increased, the nurse would discuss with the treating physician if conventional anti-emetics would be administered (Fig. 1).

### Study outcomes

The primary outcome was the number of patients that received symptomatic treatment for their nausea in the ED. Secondary outcomes were the time from inclusion to symptomatic treatment, the number of administrations of a conventional anti-emetic per patient during their stay in the ED, the estimated cost of symptomatic nausea treatment per patient, and the experiences of nurses with the use of IPA. To calculate the cost of symptomatic treatment, we used drug prices as provided by a governmental institution [10] and calculated the average cost per administered dose of conventional anti-emetic, amounting to 2.65€/dose. As all patients received 3 swabs, and no patient used more than 3 swabs, the cost of treatment with IPA was determined at 0.09€/patient (0.03€/IPA swab). A survey was distributed amongst ED nurses to evaluate their experiences with IPA inhalation. Nurses’ experiences were gauged using a survey containing 30 questions which in turn related to eight recommended outcome measures for implementation research: acceptability, adoption, appropriateness, cost, feasibility, fidelity, penetration, and sustainability [11]. All questions were asked using a five-point Likert-scale.

### Statistical analysis

We used Gpower 3.1.9.2 to calculate the sample size to detect a 20% difference in the percentage of patients receiving symptomatic treatment with type I error 0.05 and type II error 0.20. We determined that we would need 93 patients in each study phase. Statistical analyses were performed in IBM SPSS Statistics version 24. For both continuous and ordinal variables, we used the Mann-Whitney *U* test. The chi-square test was used to compare percentages. Regression analysis was used to control for confounding factors. The variables from Table 1 (age, sex, initial nausea score, initial pain score, and presumed nausea cause) were used one by one for this analysis. A *p* value <0.05 was considered as significant for all inferential tests. For the survey, we calculated an average score per question and per implementation outcome measure based on the 5-point Likert scale. Due to the limited number of surveys completed by the ED nurses, we report averages but do not make any statistical inferences based on this survey.

**Table 1:** Patient baseline characteristics

Variables	Baseline phase (***n***=106)	IPA implementation phase (***n***=104)	***p*** value
Age; median (IQR), years	50.0 (39)	57.5 (38)	0.166*
Female sex, %	57.5	66.3	0.189†
Initial nausea score; median (IQR), VAS	6 (3)	7 (3)	0.208*
Initial pain score; median (IQR), VAS	5 (6)	5 (4)	0.977*
Presumed nausea cause upon ED presentation			0.079†
Medication side effect; *n* (%)	7 (6.6)	7 (6.7)	
Abdominal pathology; *n* (%)	52 (49.1)	53 (51.0)	
Headache; *n* (%)	10 (9.4)	16 (15.4)	
Vertigo; *n* (%)	4 (3.8)	10 (9.6)	
Other; *n* (%)	33 (31.1)	18 (17.3)	

*VAS* visual analog scale, *IQR* interquartile range

^*^Mann-Whitney

^†^Pearson chi-square

## Results

### Patient characteristics

A total of 210 patients were included in the study: 106 in the baseline phase and 104 in the implementation phase. There were no significant differences between the two groups with regard to age, sex, initial nausea score, pain score, and presumed cause of nausea upon presentation in the ED (Table 1). The most commonly presumed cause of nausea was abdominal pathology (52%). Additionally, there were no significant differences between the two groups with regard to the definitive diagnosis as made by the treating physician during the ED visit (see Additional files, table A1).

### Main results

The percentage of nauseous patients that received nausea treatment was 66.0% in the baseline phase and 97.1% during the implementation phase (*p*<0.001). Additionally, the time between an entry in the ED and nausea treatment initiation fell significantly after the implementation of IPA inhalation. During the baseline phase, the median time until symptomatic treatment was 7 min (IQR 10) versus 1 min (IQR 5) during the implementation phase (*p*<0.001) (Table 2). The mean number of administered conventional anti-emetics decreased from 0.52 per patient to 0.23 administrations per patient after IPA inhalation was implemented. Comparison of the cost of nausea treatment between the two phases showed a reduction from 1.33 euro per patient to 0.67 euro per patient. Regression analysis showed that none of the included variables (age, sex, VAS pain, VAS nausea, and presumed cause of nausea) influenced our primary and secondary outcomes. A number of patients (*n*=19 for the baseline phase and *n*=11 for the implementation phase) had received conventional anti-emetic medications from the paramedics prior to arrival in the ED. Therefore, we performed a subgroup analysis for the group of patients that had not received prehospital anti-emetics. Results of this subgroup analysis were consistent with the results presented in Table 2: all significant differences in primary outcomes were also found in this separate subgroup (see Additional files, Table A2). A total of 8 patients that received IPA inhalation reported mild side effects. Three patients noted a headache, 1 patient reported dizziness, and 1 patient experienced a chemical taste. In 3 cases, nausea increased after IPA inhalation. No side effects were reported in the baseline phase.

**Table 2:** Primary outcomes based on all patients included in the study

Outcome	Baseline phase (***n***=106)	IPA implementation phase (***n***=104)	***p***-value
Patients receiving anti-emetic treatment in the ED; mean (%), number	66.0 (70)	97.1 (101)	<0.001†
Time to treatment; median (IQR), minutes	7 (10)	1 (5)	<0.001*
Administration of conventional anti-emetics in the ED; mean (95% CI), number of administrations/patient	0.52 (0.43 – 0.63)	0.23 (0.14 – 0.32)	<0.001*
Cost (Euro)	1.33	0.67	

*IQR* interquartile range, *CI* confidence interval

^*^Mann-Whitney

^†^Pearson chi-square

### Survey results

We used a survey to assess our ED nurses’ experience with the implementation of the IPA protocol. A total of 19 nurses completed the questionnaire, resulting in a response rate of 61%. Regarding the 8 implementation outcome measures, feasibility and sustainability received the highest average scores (4.11 and 4.07, respectively). Specifically, within the feasibility aspect, nurses indicated that the most important advantage of IPA inhalation was that it is easy to use (4.63) and that IPA saves them time compared to administration of conventional anti-emetics (4.47) (see Additional files, Tables A3 and A4, questions 13 and 14). Additionally, they experienced that giving inhalation instructions was easy (4.00) and instructing cost little time (4.00) (Additional files, Tables A3 and A4, questions 17 and 18). Even though the sustainability aspect consisted of only one question, nurses strongly agreed they would be glad to continue to use IPA inhalation treatment after the termination of the study (4.11) (Additional files, Table A3 and A4, question 30). Moreover, the implementation outcome measures adoption, appropriateness, and fidelity were also scored more positively than negatively (3.44, 3.39, and 3.36, respectively). Full survey data on individual questions as well as on the 8 implementation outcome measures are provided in the Additional files (see Tables A3 and A4).

## Discussion

In this study, we evaluated the practical implications of the implementation of IPA as a first-line nausea treatment in the ED. Both objective and subjective measures suggest that definitive implementation and permanent use of IPA as a first-line treatment in the ED is both viable and practically feasible. Firstly, after IPA was introduced, the number of patients receiving anti-emetic treatment rose significantly. This is most likely explained by several factors: IPA has a high user-friendliness for both the nurse and patient involved, IPA can be initiated by the nurse independently without prior permission of the treating physician, and IPA can be applied more rapidly and easily as no intravenous access is required and providing usage instructions does not require much time or effort. Secondly, the time patients had to wait for nausea treatment to be initiated was significantly reduced. Thirdly, when IPA inhalation was used, less conventional anti-emetics (Ondansetron and Metoclopramide) were prescribed and therefore the cost for symptomatic treatment of nausea in the ED dropped notably. Moreover, nurses scored seven out of eight implementation measures for IPA adoption as neutral or positive, with the feasibility aspect and sustainability aspect of IPA implementation receiving the highest scores.

The reported findings suggest that the introduction of IPA could lead to an increase in the quality of care, mainly because more nauseous patients are treated and nausea treatment is initiated more rapidly. Quality of care is also positively influenced because the valuable time freed for nurses due to IPA adoption can instead be used for other nursing activities. This was affirmed by our ED nurses as shown by the survey. Furthermore, the reduction in conventional anti-emetics associated with the introduction of IPA may also cut back on the incidence of potential adverse side effects related to these conventional medications [4]. With regard to side effects, our data shows that very few patients experienced adverse effects associated with IPA inhalation. This corresponds to previous studies [2, 7, 8]. Three patients reported an increase in nausea after IPA. However, it is questionable whether this increase was really caused by IPA or whether this can be ascribed to the natural course of the nausea.

The effectiveness of IPA as inhalation treatment against nausea in the ED was demonstrated by previous trials [7, 8]. The current study complements these earlier findings by showing for the first time the practical benefits of implementing IPA in an ED setting, considering both the patients’ but also the nurses’ perspective. The latter perspective is especially important since nurses play an essential part in the correct administration of IPA, and more generally speaking, support amongst nurses is essential for successfully implementing new treatment standards [12, 13]. Lastly, ever increasing health care costs are a daunting challenge to many health care systems [14, 15] and consequently improving health care efficiency is a present-day health care issue [16, 17]. The findings presented here can potentially encourage other EDs to install care improvement measures like IPA as nausea is a very common complaint in the ED [1, 3], and the IPA adoption seems a simple and elegant way of contributing to the reduction of health care costs while concurrently improving the quality of care.

### Limitations

Our study has a number of limitations. First of all, this was a single-center study in a peripheral hospital. Therefore, our results can potentially only be generalized to similar care settings. Secondly, the practical aspect of this study favored a before-and-after design, rather than a randomized controlled trial. A before-and-after design could lead to an overestimation of the benefit of the treatment under investigation. However, since we used a prospective control group instead of uncontrolled historical controls, we suspect that the extent to which the IPA benefits are overestimated is limited. Moreover, the observed differences in primary study outcomes between the two study phases are very large. Therefore, even in the case the results are overestimated, the implications of our results will most likely remain valid. Also, in the light of the recent attention for improvements in healthcare efficiency, implementation research like the current study contributes to the assessment of potential improvements, despite their vulnerable design. Thirdly, the higher percentage of patients receiving anti-emetic treatment and the shorter time from inclusion to treatment in the implementation phase could also at least be partially caused by the fact that the nurses had the power to decide to administer IPA. However, we feel that this is not a shortcoming of the study setup, but a clear practical advantage of IPA leading to more and faster treatment. Lastly, our research population also included patients that had already received anti-emetic treatment before presentation in our ED. This may lead to bias in the results as prehospital treatment may influence the administration of anti-emetics in the ED. However, subgroup analysis of those patients that did not receive anti-emetics from the paramedics showed that the primary study outcomes in this subgroup did not differ from the outcomes based on analysis for all patients in the study. Therefore, the potential biasing effect of prehospital treatment seems limited and hence we suggest that IPA should be applied for all nauseous patients in the ED, regardless of whether they were treated prehospitally or not.

## Conclusion

In this prospective study, implementation of IPA as the first-line treatment of nausea in the ED was associated with an increase in quality of care as more patients received treatment and nausea treatment was initiated more rapidly after IPA inhalation was introduced. Additionally, treatment cost dropped notably, suggesting that IPA implementation improves care efficiency without sacrificing quality of care. These objective findings combined with the positive ED nurse experience related to the use of IPA advocates that definitive implementation and permanent use of IPA are both viable and practically feasible. We therefore propose that IPA as the first-line nausea treatment in the ED comprises a supported and effective efficiency measure that should be more widely implemented.

## Supplementary Information

**Additional file 1: Table A1.** Definitive diagnosis as established in the emergency department.

**Additional file 2: Table A2.** Subgroup analysis of primary study outcomes for those patients that did not receive anti-emetics from the paramedics before reaching the hospital.

**Additional file 3: Table A3.** Questions included in the survey distributed amongst emergency department nurses to assess the implementation research outcomes.

**Additional file 4: Table A4.** Results of the survey evaluating the implementation research outcome measures, based on emergency department nurses’ experience with the use of IPA.

## Data Availability

The datasets used and/or analyzed during the current study are available from the corresponding author on reasonable request.

## References

[1] Myer PA, Mannalithara A, Singh G, Singh G, Pasricha PJ, Ladabaum U (2013). Clinical and economic burden of emergency department visits due to gastrointestinal diseases in the United States. Am J Gastroenterol.

[2] Egerton-Warburton D, Meek R, Mee MJ, Braitberg G (2014). Antiemetic use for nausea and vomiting in adult emergency department patients: randomized controlled trial comparing ondansetron, metoclopramide, and placebo. Ann Emerg Med.

[3] Singer AJ, Garra G, Thode HC (2016). Oligoantiemesis or inadequate prescription of antiemetics in the emergency department: a local and national perspective. J Emerg Med.

[4] Furyk JS, Meek RA, Egerton-Warburton D. Drugs for the treatment of nausea and vomiting in adults in the emergency department setting. Cochrane Database Syst Rev. 2015. 10.1002/14651858.CD010106.pub2. 10.1002/14651858.CD010106.pub2 PMC6517141 26411330

[5] Singer AJ, Garra G, Thode HC (2017). Do emergency department patients with nausea and vomiting desire, request, or receive antiemetics. Am J Emerg Med.

[6] Hines S, Steels E, Chang A, Gibbons K. Aromatherapy for treatment of postoperative nausea and vomiting. Cochrane Database Syst Rev. 2018. 10.1002/14651858.CD007598.pub3. 10.1002/14651858.CD007598.pub3 PMC6494172 29523018

[7] Beadle KL, Helbling AR, Love SL, April MD, Hunter CJ (2016). Isopropyl alcohol nasal inhalation for nausea in the emergency department: a randomized controlled trial. Ann Emerg Med.

[8] April MD, Oliver JJ, Davis WT, Ong D, Simon EM, Ng PC (2018). Aromatherapy versus oral ondansetron for antiemetic therapy among adult emergency department patients: a randomized controlled trial. Ann Emerg Med.

[9] Meek R, Egerton-Warburton D, Mee MJ, Braitberg G (2015). Measurement and monitoring of nausea severity in emergency department patients: a comparison of scales and exploration of treatment efficacy outcome measures. Acad Emerg Med.

[10] ZorginstituutNederland. www.medicijnkosten.nl [Internet]. Available from: https://www.medicijnkosten.nl/ Accessed 4 May 2020.

[11] Proctor E, Silmere H, Raghavan R, Hovmand P, Aarons G, Bunger A (2011). Outcomes for implementation research: conceptual distinctions, measurement challenges, and research agenda. Adm Policy Ment Health Ment Health Serv Res.

[12] Alvarado K (2007). Factors influencing implementation of medical directives by registered nurses: the experience of a large Ontario teaching hospital. Nurs Leadersh.

[13] Twigg DE, Duffield C, Evans G (2013). The critical role of nurses to the successful implementation of the National Safety and Quality Health Service Standards. Aust Health Rev.

[14] La Maisonneuve CD, Martins JO (2013). Public spending on health and long-term care: a new set of projections. OECD Econ Policy Pap.

[15] Emanuel EJ (2018). The real cost of the US health care system. JAMA..

[16] Hussey PS, de Vries H, Romley J, Wang MC, Chen SS, Shekelle PG (2009). A systematic review of health care efficiency measures. Health Serv Res.

[17] Cassel CK, Brennan TE (2007). Managing medical resources. JAMA.

