# Supplementary material for: Implementation of isopropyl alcohol (IPA) inhalation as the first-line treatment for nausea in the emergency department: practical advantages and influence on the quality of care
Source: Int J Emerg Med. 2021 Feb 24;14:15. doi: 10.1186/s12245-021-00334-z (PMC7905555; doi:10.1186/s12245-021-00334-z)
Supplement: Supplementary file 1 — Additional file 1: Table A1. Definitive diagnosis as established in the emergency department. [file 12245_2021_334_MOESM1_ESM.docx]

***Table A1.*** *Definitive diagnosis as established in the emergency department.*

|  | **Baseline phase (n=106)** | **IPA implementation phase (n=104)** | 0.214† |
| --- | --- | --- | --- |
| Intoxication or medication side effect; n (%) | 4 (3.8) | 5 (4.8) |  |
| Gastro-intestinal pathology; n (%) | 35 (33.0) | 45 (43.3) |  |
| Neurological pathology; n (%) | 16 (15.1) | 22 (21.2) |  |
| Metabolic derangement; n (%) | 5 (4.7) | 2 (1.9) |  |
| Urological pathology; n (%) | 9 (8.5) | 10 (9.6) |  |
| Gynaecologic pathology; n (%) | 4 (3.8) | 1 (1.0) |  |
| Trauma; n (%) | 11 (10.4) | 8 (7.7) |  |
| Other (other infections, malignancies); n (%) | 22 (20.8) | 11 (10.6) |  |

*† Pearson Chi-Square*
