# Supplementary material for: Implementation of isopropyl alcohol (IPA) inhalation as the first-line treatment for nausea in the emergency department: practical advantages and influence on the quality of care
Source: Int J Emerg Med. 2021 Feb 24;14:15. doi: 10.1186/s12245-021-00334-z (PMC7905555; doi:10.1186/s12245-021-00334-z)
Supplement: Supplementary file 2 — Additional file 2: Table A2. Subgroup analysis of primary study outcomes for those patients that did not receive anti-emetics from the paramedics before reaching the hospital. [file 12245_2021_334_MOESM2_ESM.docx]

***Table A2****: Subgroup analysis of primary study outcomes for those patients that did not receive anti-emetics from the paramedics before reaching the hospital.*

| **Primary outcome** | **Baseline phase (n=87)** | **IPA implementation phase (n=93)** | **p-value** |
| --- | --- | --- | --- |
| Patients receiving anti-emetic treatment in the ED;  percentage, (number) | 58.6 (51) | 96.8 (90) | <0.001† |
| Time to treatment; median (interquartile range), minutes | 7 (10) | 1(5) | <0.001* |
| Administration of conventional anti-emetics in the ED; mean (95% CI), amount/patient | 0.60 (0.49 – 0.71) | 0.26 (0.16 – 0.35) | <0.001* |
| Cost (euro) | 1.59 | 0.78 |  |

* Mann-Whitney
† Pearson Chi-Square
