# Supplementary material for: Implementation of isopropyl alcohol (IPA) inhalation as the first-line treatment for nausea in the emergency department: practical advantages and influence on the quality of care
Source: Int J Emerg Med. 2021 Feb 24;14:15. doi: 10.1186/s12245-021-00334-z (PMC7905555; doi:10.1186/s12245-021-00334-z)
Supplement: Supplementary file 3 — Additional file 3: Table A3. Questions included in the survey distributed amongst emergency department nurses to assess the implementation research outcomes. [file 12245_2021_334_MOESM3_ESM.docx]

***Table A3:*** *Questions included in the survey distributed amongst emergency department nurses to assess the implementation research outcomes. Questions consisted of a statement that was to be rated on a 5-point Likert scale, ranging from 1=strongly disagree to 5=strongly agree.*

| **Outcome** | **No.** | **Question** |
| --- | --- | --- |
| **Acceptability** | 1 | Patients did not doubt the effect of IPA prior to IPA administration |
|  | 2 | Patients regard IPA as an acceptable treatment option |
|  | 3 | I regard IPA as an acceptable treatment option |
|  | 4 | Patients rather take medications than inhale IPA |
| **Adoption** | 5 | Prior to the study I had confidence in the effectiveness of IPA |
|  | 6 | Prior to the study I saw IPA as an added value to our ED |
|  | 7 | Prior to the study I thought IPA would save me time |
|  | 8 | Prior to the study I had confidence in a successful implementation of IPA in our ED |
|  | 9 | Prior to the study I was enthusiastic about having to work with IPA |
| **Appropriateness** | 10 | I think IPA is an effective treatment against nausea |
|  | 11 | I think IPA is a better option to treat nausea than Zofran/Primperan |
| **Cost** | 12 | I start an IV less frequently in nauseous patients since I started to use IPA |
| **Feasibility** | 13 | IPA is easy to use |
|  | 14 | IPA saves me time compared to Zofran/Primperan |
|  | 15 | The time a nauseous patient has to wait before nausea treatment is initiated is shorter after IPA implementation |
|  | 16 | Because of the use of IPA people suffering from mild nausea also receive treatment |
|  | 17 | Instructing patients on the use of IPA is easy |
|  | 18 | Instructing patients on the use of IPA costs little time |
|  | 19 | Patients adhere very well to the IPA instructions |
|  | 20 | Patients seem satisfied with IPA treatment |
|  | 21 | Generally speaking, IPA is of added value in our ED |
|  | 22 | IPA is a good first-line treatment against nausea |
| **Fidelity** | 23 | I always adhere to the protocol for anti-emetic treatment |
|  | 24 | When IPA does not seem to have the desirable effect after 3 swabs, I sometimes give patients extra swabs before switching to Zofran/Primperan |
|  | 25 | When IPA does not seem to work after 1 swab, I preferably give Zofran/Primperan immediately |
|  | 26 | Also in cases where IPA seems to relieve nausea, I preferably give Zofran/Primperan immediately |
|  | 27 | I regularly skip IPA as a first-line treatment as I prefer to start with Zofran/Primperan as the first step |
|  | 28 | I sometimes provide patients with more swabs than the IPA protocol prescribes |
| **Penetration** | 29 | I use the IPA protocol for all eligible nauseous patients in the ED |
| **Sustainability** | 30 | After terminating this study, I would like to continue to use IPA for the treatment of nausea |
