# Supplementary material for: Implementation of isopropyl alcohol (IPA) inhalation as the first-line treatment for nausea in the emergency department: practical advantages and influence on the quality of care
Source: Int J Emerg Med. 2021 Feb 24;14:15. doi: 10.1186/s12245-021-00334-z (PMC7905555; doi:10.1186/s12245-021-00334-z)
Supplement: Supplementary file 4 — Additional file 4: Table A4. Results of the survey evaluating the implementation research outcome measures, based on emergency department nurses’ experience with the use of IPA. [file 12245_2021_334_MOESM4_ESM.docx]

***Table A4:*** *Results of the survey evaluating the implementation research outcome measures, based on emergency department nurses’ experience with the use of IPA.*

| **Outcome** | **No.** | **Answer on the Likert scale** | | | | | | **Avg per question** | | **Avg per outcome measure** |
| --- | --- | --- | --- | --- | --- | --- | --- | --- | --- | --- |
|  |  | **1** | **2** | **3** | **4** | **5** |  | |  | |
| **Acceptability** | 1 | 0 | 7 | 7 | 5 | 0 | 2,89 | | 3,05 | |
|  | 2 | 0 | 3 | 10 | 6 | 0 | 3,16 | |  | |
|  | 3 | 0 | 1 | 8 | 7 | 3 | 3,63 | |  | |
|  | 4 | 2 | 8 | 6 | 3 | 0 | 2,53 | |  | |
| **Adoption** | 5 | 0 | 1 | 13 | 5 | 0 | 3,21 | | 3,44 | |
|  | 6 | 0 | 0 | 7 | 12 | 0 | 3,63 | |  | |
|  | 7 | 0 | 2 | 6 | 10 | 1 | 3,53 | |  | |
|  | 8 | 0 | 0 | 10 | 9 | 0 | 3,47 | |  | |
|  | 9 | 0 | 1 | 10 | 8 | 0 | 3,37 | |  | |
| **Appropriateness** | 10 | 0 | 2 | 6 | 7 | 4 | 3,68 | | 3,39 | |
|  | 11 | 0 | 5 | 8 | 5 | 1 | 3,11 | |  | |
| **Cost** | 12 | 3 | 7 | 4 | 3 | 2 | 2,68 | | 2,68 | |
| **Feasibility** | 13 | 0 | 0 | 1 | 5 | 13 | 4,63 | | 4,07 | |
|  | 14 | 0 | 0 | 2 | 6 | 11 | 4,47 | |  | |
|  | 15 | 0 | 1 | 1 | 11 | 6 | 4,16 | |  | |
|  | 16 | 0 | 0 | 3 | 13 | 3 | 4,00 | |  | |
|  | 17 | 0 | 1 | 0 | 16 | 2 | 4,00 | |  | |
|  | 18 | 0 | 1 | 1 | 14 | 3 | 4,00 | |  | |
|  | 19 | 0 | 0 | 6 | 11 | 2 | 3,79 | |  | |
|  | 20 | 0 | 1 | 4 | 14 | 0 | 3,68 | |  | |
|  | 21 | 0 | 0 | 3 | 13 | 3 | 4,00 | |  | |
|  | 22 | 0 | 0 | 4 | 12 | 3 | 3,95 | |  | |
| **Fidelity** | 23 | 0 | 7 | 6 | 5 | 1 | 3,00 | | 3,36 | |
|  | 24 | 2 | 12 | 2 | 3 | 0 | 2,32 | |  | |
|  | 25* | 0 | 6 | 3 | 7 | 3 | 3,37 | |  | |
|  | 26* | 0 | 1 | 4 | 11 | 3 | 3,84 | |  | |
|  | 27* | 0 | 3 | 5 | 8 | 3 | 3,58 | |  | |
|  | 28* | 0 | 0 | 3 | 12 | 4 | 4,05 | |  | |
| **Penetration** | 29 | 0 | 4 | 10 | 5 | 0 | 3,05 | | 3,05 | |
| **Sustainability** | 30 | 0 | 1 | 2 | 10 | 6 | 4,11 | | 4,11 | |
| * Answers to these questions have been reversed in order to ensure comparability with remaining questions, where 5 is the most positive outcome and 1 the most negative outcome. | | | | | | | | | | |
